# All eyes on PCS: analysis of the retinal microvasculature in patients with post-COVID syndrome—study protocol of a 1 year prospective case–control study

**DOI:** 10.1007/s00406-023-01724-5

**Published:** 2023-12-02

**Authors:** Timon Kuchler, Renate Hausinger, Matthias C. Braunisch, Roman Günthner, Rebecca Wicklein, Benjamin Knier, Nathalie Bleidißel, Matthias Maier, Andrea Ribero, Maciej Lech, Kristina Adorjan, Hans Stubbe, Konstantin Kotliar, Uwe Heemann, Christoph Schmaderer

**Affiliations:** 1 Department of Nephrology, Klinikum rechts der Isar, School of Medicine, Technical University of Munich, Ismaninger Str. 22, 81675 Munich, Germany; 2 Department of Neurology, Klinikum rechts der Isar, School of Medicine, Technical University of Munich, Ismaninger Str. 22, 81675 Munich, Germany; 3 Department of Ophthalmology, Klinikum rechts der Isar, School of Medicine, Technical University of Munich, Ismaninger Str. 22, 81675 Munich, Germany; 4 Medizinische Klinik und Poliklinik IV, LMU University Hospital Munich, Ziemssenstraße 5, 80336 Munich, Germany; 5 Department of Psychiatry and Psychotherapy, LMU University Hospital Munich, Nußbaumstraße 7, 80336 Munich, Germany; 6 Medizinische Klinik und Poliklinik II, LMU University Hospital Munich, Marchioninistraße 15, 81377 Munich, Germany; 7 Aachen University of Applied Sciences, Heinrich-Mussmann-Str. 1, 52428 Jülich, Germany

**Keywords:** Post-COVID syndrome, Long-COVID, SARS-CoV-2, COVID-19, Endothelial dysfunction, Retinal microvasculature

## Abstract

Since widespread vaccination against COVID-19, the development of effective antiviral drugs, and the decreasing number of patients with COVID-19 in intensive care, the risk from SARS-CoV-2 infection appears less threatening. However, studies show that a significant number of patients suffer from long-term sequelae, even months after SARS-CoV-2 infection. The so-called post-COVID syndrome (PCS) often presents a diagnostic and treatment challenge for physicians. This study protocol describes the “All Eyes on PCS” study, which aims to investigate the retinal microvasculature in PCS patients and COVID-19-recovered patients to provide new insights into the pathophysiology of PCS. “All Eyes on PCS” is a prospective, case–control study with the primary objective of detecting endothelial dysfunction (ED) in patients with PCS. Therefore, we intend to recruit patients with PCS, fully SARS-CoV-2-infection-recovered (CR) participants, and SARS-CoV-2-infection-naïve (CN) participants. Baseline measurements will include: (1) patient-specific characteristics, (2) biochemistry, (3) retinal vessel analysis (RVA), (4) survey questionnaires as patient-reported outcomes measurements (PROMs), (5) optical coherence tomography (OCT), OCT angiography (OCTA), and adaptive optics (AO), (6) blood pressure recordings, (7) handgrip strength test. After 6 months, baseline measurements will be repeated in the PCS cohort, and after 1 year, a telephone query will be conducted to assess residual symptoms and treatment needs. The aim of this study is to gain insight into the pathophysiology of PCS and to provide an objective biomarker for diagnosis and treatment, while also creating a comprehensive clinical database of PCS patients.

ClinicalTrials.gov Identifier: NCT05635552; Date: 2.12.2022.

**Supplementary Information:**

The online version contains supplementary material available at 10.1007/s00406-023-01724-5.

## Background and rationale

### Introduction

COVID-19 is a systemic disease that may result in hyperinflammation caused by infection with the severe acute respiratory syndrome coronavirus 2 (SARS-CoV-2). In the acute phase of severe disease, hyperinflammation is associated with thromboembolism, myocarditis, disseminated intravascular coagulation, and subsequent respiratory distress syndrome [1]. Although the lungs are primarily affected in this stage, SARS-CoV-2 can also cause systemic endothelial dysfunction and inflammation [2].

Endothelial dysfunction (ED) in acute SARS-CoV-2 infection is characterized by elevated markers of endothelial damage and coagulopathy, and SARS-CoV-2-RNA has been directly detected in the endothelium of deceased patients [3–6]. The angiotensin-converting enzyme-2 (ACE-2) receptor, which serves as the entry point of SARS-CoV-2 into cells, is expressed not only in various tissues but also in endothelial cells [7, 8]. There is also evidence that the ACE-2 receptor and transmembrane protease serine subtype 2 (TMPRSS2) are expressed in retinal cells, which may facilitate viral internalization into the cell [9, 10]. Studies have shown that infection with the viral S1 subunit leads to endothelial damage, cytokine release, and thrombosis [11, 12]. Internalization of SARS-CoV-2 into the cell and retention in the endosome may lead to Toll-like receptor (TLR)-dependent activation of the NF-κB pathway, resulting in changes in the expression of various genes that could contribute to ED [13]. Whether ED is directly caused by the virus or only indirectly through systemic inflammation remains unclear [14].

While most of SARS-CoV-2-infected individuals fully recover after several weeks, symptoms such as shortness of breath, chest pain, cognitive dysfunction, fatigue, and palpitations persist for months in several patients, as do psychosocial symptoms such as depression, sleep disturbances, and agitation [15]. This is commonly referred to as post-COVID syndrome (PCS, also known as Long-COVID, post-COVID condition), which the WHO defines as a complex of complaints following SARS-CoV-2 infection, usually occurring 3 months after the onset of COVID-19 with symptoms lasting at least 2 months and which cannot be explained by any other diagnosis [16]. In most cases, developing after a mild to moderate primary infection, PCS has an estimated prevalence of around 12–35%. It is important to distinguish from post-intensive care syndrome (PICS), which refers to long-term physical, cognitive, and psychological symptoms that can occur after treatment of severe SARS-CoV-2 infection in intensive care unit (ICU) [17–19].

The pathophysiology and mechanisms leading to PCS are still unclear. One possible explanation is that ED persists longer in patients than the acute infection and contributes to chronic, ongoing post-infectious symptoms [20–22]. One common symptom in patients with PCS is persistent fatigue, which often fulfills diagnostic criteria of myalgic encephalomyelitis/chronic fatigue syndrome (ME/CFS) [23, 24]. Patients with ME/CFS also show ED which affects both small and large vessels [25]. Immunological abnormalities due to chronic virus persistence may lead to inflammation, and therefore to a prolonged disturbance of the fine interplay between endothelium, immune cells, and platelets [26, 27].

Although some patients with PCS exhibit organ-related involvement, such as myocarditis detected by MRI, elevated troponin T levels, or reduced pulmonary diffusion capacity, there is currently no established routine parameter for characterizing PCS [28]. Endothelial function seems to improve after acute SARS-CoV-2 infection; however, it is still detectable via flow-mediated dilation (FMD) after 6 months [29]. There is evidence suggesting that persistent ED is an independent marker of developing PCS and preliminary findings suggest that the retinal microcirculation may also be affected following severe SARS-CoV-2 infection [30, 31]. However, to our knowledge, until now retinal vessel analysis (RVA) has not been used in PCS patients to characterize ED.

Our hypothesis is that patients with PCS suffer from a prolonged ED which leads to typical PCS symptoms such as fatigue, dyspnea, and cognitive dysfunctions. To examine the responsiveness of retinal vessels to flickering light as a surrogate for ED, we will use RVA and to further characterize the retinal microcirculation, we will include OCT angiography (OCTA) and adaptive optics (AO) measurements. With the study “All Eyes on PCS”, we want to bring new insights into the pathophysiology and possible risk factors of prolonged ED in PCS patients. An impaired retinal microcirculation might prove as a strong and independent predictor of PCS, and therefore serve as an objective biomarker in diagnosis and therapy monitoring. Our objective is to establish a comprehensive biobank comprising individuals with PCS and those who have recovered from COVID-19, with the aim of investigating the pathophysiological mechanisms underlying PCS. The biobank will serve as a valuable resource aimed at understanding the molecular, cellular, and immunological aspects of PCS and help identify potential targets for intervention.

### Objectives

#### Research hypothesis

Patients with PCS exhibit prolonged ED when compared with a SARS-CoV-2-infection-recovered (CR) cohort and SARS-CoV-2-infection-naïve (CN) cohort. This prolonged ED is accompanied by a range of PCS-specific complaints, including fatigue, palpitations, and cognitive impairment. We hypothesize that the symptom burden of PCS is associated with an impaired retinal vessel responsiveness and changes in vessel morphology.

The primary objective of our study is to quantify retinal vessel responsiveness and vessel morphology using the Retinal Vessel Analyzer (RVA) in patients with PCS and compare these parameters to those of the CR and CN cohort.

Secondary objectives are:

1. To collect patient-reported symptoms (PROMs), assess PCS symptom severity, and then correlate these findings with RVA parameters at baseline.
2. To monitor the development of symptom severity of PCS and endothelial function after 6 months in the PCS cohort.
3. To conduct an in-depth study of the retina microcirculation using OCTA and AO.
4. To classify patients based on the different organ systems affected by PCS and compare RVA parameters between these groups.
5. To quantify fatigue in patients using the handgrip strength test as a measure of excessive fatiguability.
6. To use cell culture experiments on retinal epithelial and endothelial cells to explain a possible phenotype of ED in PCS patients.
7. To characterize the immune phenotype of T cell and monocyte subpopulations in PCS patients.
8. To investigate the potential contribution of virus reactivation, specifically Epstein–Barr virus (EBV), to ED in PCS patients.
9. To establish a possible link between ED and the development of autonomic dysfunction by measuring basal cortisol levels in PCS patients.

## Trial design

### Methods: participants, measurements, and outcomes

Data collection will take place in the University Hospital of the Technical University (TU) Munich, Klinikum rechts der Isar, Germany. Three departments are involved in data collection: Department of Nephrology (lead), Department of Neurology, and Department of Ophthalmology. Recruitment of patients will take place in the post-COVID outpatient clinic at the University Hospital of the Ludwigs-Maximilians-University (LMU) Munich, Campus Großhadern, in general practitioners and via social media.

Inclusion criteria:

i. Patients with PCS (positive PCR or positive rapid antibody test ≥3 months) with a currently existing, PCS-typical complaint complex, ongoing for at least 2 months that cannot be explained by an alternative diagnosis.
ii. Control group (CR): participants recovered from SARS-CoV-2 infection (positive PCR or positive rapid antibody test ≥ 3 months) without any residual symptoms.
iii. Healthy cohort (CN): no history of SARS-CoV-2 infection (exclusion via measurement of disease specific antibodies).

Exclusion criteria:

i. Missing or incomplete consent form.
ii. Age < 18 years.
iii. Pregnancy.
iv. Malignancy.
v. Diseases associated with a significant change in life expectancy.
vi. Autoimmune diseases of the rheumatological type.
vii. Cataract.
viii. Epilepsy.
ix. Glaucoma.

Measurements are carried out by experienced examiners in the departments of nephrology, neurology and ophthalmology. Informed consent is obtained by a clinical study investigator prior to inclusion. A structured medical history and the entry criteria are re-evaluated. A written consent form approved by the ethics committee of the Technical University of Munich, School of Medicine, is used to obtain informed consent.

To establish a PCS biobank, we ask patients to donate their biospecimens. For this purpose, we use an information sheet approved by the ethics committee of the Technical University of Munich, School of Medicine.

### Outcomes

#### Primary outcome

PCS patients show an impaired retinal vessel responsiveness and microcirculation when compared to a SARS-CoV-2-infection-recovered cohort. To investigate ED, both parameters (vMax and aMax) are important [32]. In a recent publication, we could show that vMax is a predictor for all-cause mortality in end-stage renal disease (ESRD) [33]. In addition, we will compare parameters of retinal microcirculation. To analyze microvascular function, central retinal artery equivalent (CRAE), central retinal vein equivalent (CRVE), and arteriolar–venular ratio (AVR) are important variables. Narrower CRAE and wider CRVE have been shown to be predictors for cardiovascular mortality [34]. Therefore, our primary endpoint will focus on differences in both dynamic retinal vessel analysis (DVA, vMax and aMax) and static retinal vessel analysis (SVA, CRAE, CRVE, and AVR) between our PCS cohort and fully SARS-CoV-2-infection-recovered patients.

Secondary outcomes:

1. To determine whether PCS patients have an impaired retinal vessel responsiveness at baseline compared to the CN cohort. DVA parameters, including aMax and vMax, will be measured at baseline, and mean or median values will be calculated for each cohort, respectively.
2. To assess whether SVA parameters of retinal vessel analysis (including CRAE, CRVE and AVR) are altered in the PCS cohort compared with CN cohorts. The baseline, mean or median values of these parameters will be calculated for each cohort, respectively.
3. To investigate whether PCS patients show an improved retinal vessel responsiveness and static parameters after 6 months compared to baseline parameters. The static and dynamic parameters will be measured at baseline and follow-up and mean or median values will be calculated for each cohort, respectively.
4. To assess whether symptom severity of PCS correlates with impaired retinal vessel responsiveness and static parameters of retinal vessel analysis. The symptom severity measured with patient-reported outcome measures (PROMs), along with the baseline static and dynamic parameters, will be assessed, and mean or median values will be calculated.
5. To examine whether patients with PCS show a chronic immune activation and a changed amount of circulating angiogenic cells (CAC) and circulating endothelial progenitor cells (CEC) in fluorescence-activated cell sorting (FACS) analysis compared with the CR and CN cohort. At baseline, percentage of CAC and CEC will be measured and mean or median values will be calculated for each cohort, respectively.
6. To assess whether epithelial and endothelial cells show a change in surface markers and inflammation markers when incubated with recombinant SARS-CoV-2 S1 subunit protein and/or patient serum from the PCS cohort, in cell culture experiments.
7. To determine whether PCS patients show elevated markers of ED (sICAM, sVCAM, Thrombomodulin, P-Selektin, ADMA, SADMA, Endothelin-1, ACE-1, ACE-2, ANG-2, Pentraxin-3, GDF-15) compared to the CR and CN cohort. At baseline, mean or median values of these markers will be calculated for each cohort, respectively.
8. To investigate whether PCS patients show elevated markers of chronic inflammation (INFß, TNFα, IFNy, Il-8, Il-6, Il-1ß, Mcp1, Il-10) compared to the CR and CN cohort. At baseline, mean or median values of these markers will be calculated for each cohort, respectively.
9. To examine whether PCS patients show a reactivation of Epstein–Barr virus (EBV). At baseline, PCR of EBV DNA will be measured and mean or median values will be calculated for each cohort, respectively.
10. To assess whether patients with PCS show changes of the retinal vasculature as measured by OCT angiography. At baseline, mean values of retinal vessel densities of the superficial and deep vascular complex as well as size of the foveal avascular zone in both eyes will be measured for each cohort, respectively.
11. To investigate whether PCS patients with impaired retinal vessel responsiveness show an autonomic dysfunction characterized by low cortisol levels. At baseline, mean or median values of cortisol levels will be measured for each cohort, respectively.
12. To explore whether PCS patients with fatigue fulfill diagnostic criteria of ME/CFS. At baseline, the Canadian Criteria of CFS score and the handgrip strength will be measured and mean or median will be calculated)

### Participant timeline

**Fig. 1:**
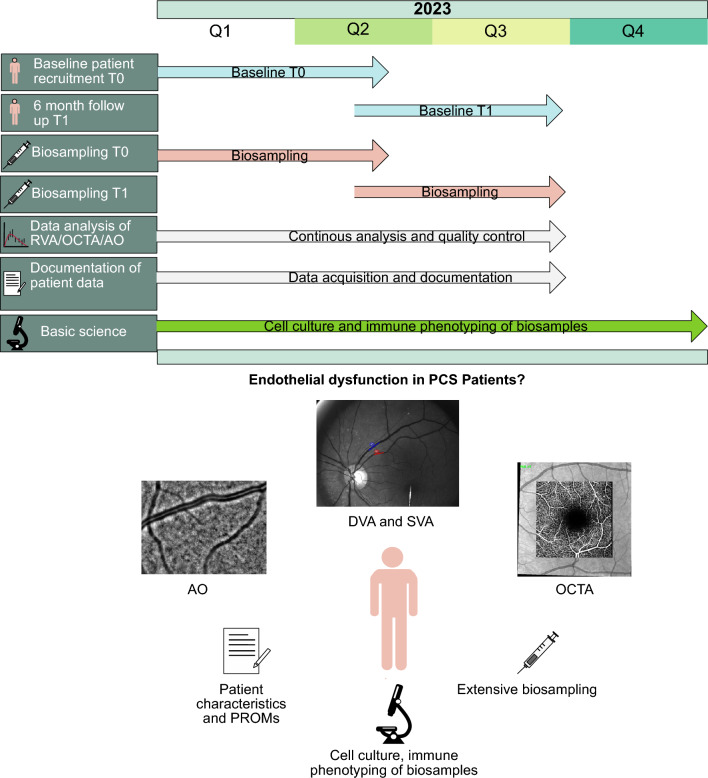
Participant timeline and overview of conducted measurements. Figure shows baseline (T0) and follow-up (T1) measurements that are conducted in the “All Eyes on PCS” study. The figure provides a visual representation of the planned measurements and includes sample images of key assessment tools, including adaptive optics (AO), dynamic and static retinal vessel analysis (DVA, SVA) and OCT angiography (OCTA), and patient reported outcome measures (PROMs)

Recruitment for our study is scheduled to begin in October 2022, with the first patients expected to be measured by the end of 2022 or early 2023. In addition to measuring the PCS cohort, CR participants will be recruited and measured, with completion of baseline measurements (T0) planned by June 2023. In the PCS cohort, measurements will be repeated after 6 months (T1), with completion planned by the end of September 2023. One year after the initial measurements, we will conduct a telephone survey to assess any residual symptoms and treatment needs. Data analysis and especially data quality control will continuously happen after measurement of the first patient. Cell culture experiments are ongoing starting with incubation of retinal endothelial cells with SARS-CoV-2 spike antigen (S1) followed by incubation with patient serum (Fig. 1).

For a detailed list of the variables to be measured, the departments involved in the study, and the relevant hypotheses, please refer to Table 1.

**Table 1:** Detailed list of measured variables

Questionaries	Domain	Item
*Psychological assessment*		
Munich COVID history questionarie (MuCOV)	Social demographics, history of primary SARS-CoV-2 infection, vaccination history, PCS symptoms (19 frequently reported symptoms), medical history, preconditions	78
FSS (Fatigue severity scale) [35]	Covering signs and symptoms of fatigue	7
C19-YRS (“COVID-19 Yorkshire Rehabilitation Scale”)	Covering PCS-specific symptoms	15
GAD-7 (“Generalized Anxiety Disorder Scale-7”) [36]	Questionnaire to evaluate symptoms of a generalized anxiety disorder	7
Chronic Fatigue Questionnaire [37]	Adapted from the Canadian criteria for the diagnosis of CF	
PHQ-9 (“Patient Health Questionaire”) [38]	Asks for symptoms of depression over the past two weeks	9

Technical measurements	Measured variables	Department
Dynamic retinal vessel analysis	Arteriolar dilation (aMax), venular dilation (vMax)	Nephrology
Static retinal vessel analysis	central retinal artery equivalent (CRAE), central retinal vein equivalent (CRVE) arteriolar/venular ratio (AVR)	Nephrology
Optical coherence tomography (OCT) angiography	Vessel density of whole image, superficial vascular complex (SVC) and deep vascular complex (DVC), foveolar avascular zone (FAZ)	Neurology
Adaptive optics	Wall to lumen ratio (WLR)	Ophthalmology
Handgrip strength test	Fatigability of the forearm muscles	Nephrology

Biochemistry	Measured variables	
Routine laboratory	K, Na, Krea, Bun-N, Protein-total, CRP, Chol, Tri, LDL, HDL, GPT, yGT, ChE, GOT, LDH, CK, Troponin, Fe, Ferritin, Transferrin, Transferrin saturation, TSH, Quick, pTT, D-Dimer, IgG subclasses (IgG 1–4), basal cortisol secretion (basal salivary cortisol secretion)	Clinical chemistry
Virus reactivation	EBV DNA	Virology
Endothelial dysfunction markers	sICAM, sVCAM, Thrombomodulin, P-Selektin, ADMA, SADMA, Endothelin-1, ACE-1, ACE-2, ANG-2, Pentraxin-3, GDF-15, VEGF	Nephrology
Chronic inflammation	INFß, TNFα, IFNy, IL-8, IL-6, CXCL-10, MCP-1,	Nephrology

Immune phenotyping	Cell line characteristics	Hypothesis
T-cell exhaustion	CD8 + and CD4 + cells expressing markers of exhaustion	Chronic immune activation
Circulating endothelial progenitor cells	Endothelial cells which are mobilized in response to ischemia and migrate to sites of endothelial damage	ED
Circulating angiogenic cells	Monocyte population with angiogenic effects	ED

The table displays a detailed list of the measured variables and the department responsible for conducting the measurements. For the immune phenotyping, the table also includes the research question addressed in the “All Eyes on PCS” study”

### Sample size

In the PCS and CR cohort, we plan to recruit 100 participants. The SARS-CoV-2-infection-naïve cohort is defined as no history of SARS-CoV-2 infection (exclusion via measurement of specific antibodies) and consists of an already established, pre-pandemic healthy cohort and a cohort recruited during the pandemic.

Primarily, a comparison of the group means regarding the dilation of the retinal vessels will be performed using an analysis of variance (ANOVA) (significance level *α* = 5%).

To estimate the required sample size, we will assume a standard deviation within groups of 2%, based on the largest normative sample published to date, which is consistent with previous healthy control studies in the “diseased vs. healthy controls” group comparison. [39]. A biologically relevant mean difference of aMax between groups is assumed to be 1%—specifically, 2.5 vs. 3.5% dilation for PCS vs. CR. Similar differences of this magnitude have been observed in comparable groups with other disease entities, such as heart failure [40]. With these assumptions, we estimate that 78 evaluable patients per group will be needed to reject the null hypothesis (equality of all group means) with a power of 90%. To account possible dropouts or measurement errors, 100 patients per group should be included in the study. If the global null hypothesis is rejected based on ANOVA, pairwise group comparisons will be made at a significance level of *α* = 5% as a final test procedure. Power analysis was done by BH using nQuery (version 9.1.0.0).

### Recruitment

To achieve an adequate recruitment number, we cooperate with the post-COVID outpatient clinics of the LMU University Hospital (KA, HS). Via social media of the Klinikum rechts der Isar, flyers will be additionally posted online and distributed. To provide adequate information for participants and patients, we are creating a website with further information (https://www.mri.tum.de/alleyesonpcs). Patients recruited via social media who wish to participate in the study must complete a screening form to ensure that only those with PCS are enrolled. Potential study participants will be discussed using the screening form in a weekly meeting with the chief investigator, PI, and doctoral students and will be included or excluded based on a majority vote. In particular, the temporal relationship between acute SARS-CoV-2 infection and the onset of PCS symptoms but also alternative diagnoses are discussed. Enrollment of participants will be conducted by a physician from the Department of Nephrology.

### Data collection and management

We have written “standard operating procedures (SOPs)” to ensure standardization of processes. SOPs were translated and can be found in the supplements. Study instruments include:

*Questionnaires:* Only standardized and established questionnaires are used to assess patient-reported outcomes (“PROMs”).

*Laboratory tests:* Routine parameters are measured by our in-house clinical chemistry department.

*DVA and SVA analysis:* Data acquisition will be performed by following the SOPs (Online Resource 1). All examiners will be trained by a single experienced supervisor and must reach high image accuracy and quality in at least ten volunteers. Examiners do not perform data analyses and are only responsible for data acquisition. Data analysis will be performed by independent scientists and will be compared afterward. One rater will perform a quality evaluation and exclude insufficient measurements. Vessel response curves will be evaluated using the cumulative scoring method. The score ranges from 0 to 5. Retinal images with insufficient quality (< 2.5) will be discussed with a second rater and then excluded.

*OCT and OCT angiography:* Measurements and data analysis are performed by the Department of Neurology (Online Resource 2).

*AO:* Measurements and data analysis are undertaken by the Department of Ophthalmology (Online Resource 3).

*Handgrip strength test:* Data acquisition is done by following the SOP “Handgrip strength test” (Online Resource 4).

To promote participant retention, we contact the participants 2 days before their follow-up appointment to ensure that they remember the follow-up visit. Detailed information of PCS patients which were lost to follow-up will be provided.

### Data management and confidentiality

Data entry only takes place in the University Hospital of the TU Munich on an assigned key-locked study computer. Excel sheets used for data collection are key-locked and standardized. All collected data will be verified by an independent second scientist (double data verification) and checked for plausibility (e.g., range checks for clinical data) (Online Resource 3). Data which are collected analog e.g., questionnaires and clarification sheets, are stored in a locked room in the University Hospital of the TU Munich. We have developed a checklist for each admitted participant to ensure all necessary information is collected, and in the event of missing data, we have established a protocol for conducting a standardized evaluation (Online Resource 3). Clinical data will be stored on a dedicated study server and in non-pseudonymized form on hard drives (accessible only to study physicians and persons directly involved in the study). Blood samples are labeled with the patient ID and will be stored in the routine nephrology laboratory and in the nephrology research laboratory in locked rooms.

### Pseudonymization

For further analysis, all patient data are undergoing double pseudonymization. Patient-related data such as age and gender will be stored in a list, with a sequential study number assigned to each patient. The clinical data are exclusively assigned to the study numbers in a separate list. Both lists are password protected. The list containing the patient-related data and study numbers is kept separately from all other lists by the study director in a password-protected computer. Clinical data and informed consent forms are kept for 10 years, respectively.

Upon revocation of informed consent, all data will be deleted and printed records and collected samples will be destroyed.

### Storage of biological specimens for genetic or molecular analysis in this trial

After recruitment, 40 ml of blood is initially collected from patients and sent directly to the nephrology laboratory. There, a portion of the blood is sent for analysis by clinical chemistry, and another portion is further processed. Peripheral blood mononuclear cells (PBMCs) are isolated from the processed blood according to protocol and stored at – 80 °C. In addition, samples of serum and whole blood are also frozen at -80 °C for future analysis; however, are not stored longer than 10 years.

### Statistical methods

All statistical analysis will be carried out using R in the current version implemented in RStudio and will be supervised by BH. Used R Packages and their version numbers will be reported in the peer-reviewed manuscripts.

We plan to match our PCS cohort with the CR recovered and CN cohort at least 1:1 based on age and gender. The success of the matching is checked by the matchbalance function and results will be published in the supplements. To create a baseline description before and after matching, Table1 function is used. Depending on the distribution of the values aMax and vMax, a parametric (*t* test) or non-parametric test (Mann–Whitney test) is used to compare the means or medians, respectively. Boxplots will be plotted with the ggplot2 function. To evaluate a possible function as a biomarker in PCS, receiver operating characteristic (ROC) curves and the area under the curve (AUC) will be calculated using the plotROC function. Correlations will be visualized with the corrplot function. In general, Spearman or Pearson coefficients are calculated depending on the relationship between the two variables. To check for confounding, we will use linear regression models. Interim analyses are planed after completion of baseline recruitment. All participant scientists will have access to the evaluated data. Data will be published after baseline evaluation and after completed follow-up. To perform subgroup analyses, a test for interaction is performed using ANOVA and then visualized using the interaction.plot function. Information and baseline characteristics of patients lost to follow-up will be compared with those who completed the study using non-parametric or parametric tests, respectively. Upon reasonable request, access to the raw data and statistical codes used will be granted to the reviewers in the peer-review process when needed. Another sharing is not intended.

### Oversight and monitoring

*Chief investigator:* Weekly meeting via Zoom with the study base team and PIs. Responsible for overseeing the trial in general.

*Principal investigator (PI)*: Responsible for organizing and coordination of different involved departments and recruitment process in PCS ambulances. Responsible for data management and storage and final data analysis.

*RVA-team:* Monthly meeting of the three supervisors of RVA (RG, KK, TK). Quality control, data management,and updating on newest developments in the trial. Maintenance of the RVA machine.

*Head of biobank:* Processing and storage of bio samples. Maintenance of databank.

*Study base team*: Responsible to carry out technical measurements and blood sample drawing. Arrangement of study appointments. Not primarily involved in data analysis.

Data management is carried out by the PI who directly reports to the chief investigator. Data verification is done by the head of biobank which also carries out data collection and maintenance.

However, in case of RVA parameters, data analysis will be carried out by three independent principal investigators (RG, KK, TK). Data are checked frequently for validity and quality. If abnormalities occur, they are discussed between these three PIs and report to the chief investigator.

If we plan to amend the study protocol (e.g., new measurement of certain parameters), we will consult the ethical review board of TU Munich to discuss the feasibility of implementation. If the amendment is feasible, we will notify all participating researchers of the change. If the change affects study participants, we will inform them of the possible change and obtain new written informed consent. There are no publication restrictions, and we will publish the results in an appropriate peer-reviewed journal.

## Discussion

The “All Eyes on PCS” study aims to investigate the potential pathophysiological mechanisms underlying a prolonged endothelial dysfunction (ED) in patients with PCS. The study will use RVA to measure the responsiveness of retinal vessels to flickering light and the retinal microcirculation as a surrogate for ED, as well as OCT and AO measurements to further characterize microvascular function. The design of this study is primarily focused on characterizing ED in PCS patients. In addition, the study aims to establish a comprehensive biobank of PCS and CR cohorts to answer questions regarding the pathophysiology of PCS.

### Limitations

To conduct a full-scale characterization of PCS patients, we anticipate challenges in coordinating between the involved departments and scientists, particularly due to the need to perform three technical measurements that require specific expertise and quality standards. To ensure a smooth process, we conducted trial runs, developed SOPs before the start of recruitment, and are using digital study calendars. As participation in the study is voluntary and no immediate benefits are expected for the participants, we anticipate a certain drop-out rate. To avoid recruiting only mildly ill PCS patients, we are collaborating with the PCS ambulance of the LMU University Hospital. However, this could introduce bias toward recruiting only severely affected PCS patients, as mildly affected patients may not make the effort to get an appointment at the ambulance. Therefore, we are adopting a broad recruitment approach that includes involvement of general practitioners and self-help groups to overcome this potential bias.

## Conclusion

This paper describes the study protocol for analyzing retinal microvasculature in patients with PCS. Using a multidisciplinary approach that involves comprehensive clinical assessments, retinal microcirculation imaging, blood and tissue sampling, a basic science approach, and patient-reported outcomes, we aim to identify potential risk factors for PCS and gain a better understanding of its pathophysiology. We anticipate that the study will lead to the identification of possible biomarkers and could help improve the management and treatment of PCS.

### Trial status

Protocol version number 2. 28.03.23. Date of recruitment start: 19.10.2022. Date of the expected end of the study: Baseline recruitment (T1): End of May 2023. Follow-up: End of September 2023.

## Supplementary Information

Below is the link to the electronic supplementary material.

Supplementary file1 (DOCX 5164 KB)

## Data Availability

All investigators involved in the planning and implementation of the study will have access to the final trial dataset.

## Abbreviations

ACE-2: Angiotensin-converting enzyme-2
ANOVA: Analysis of variance
AO: Adaptive optics
AUC: Area under the curve
AVR: Arteriolar–venular ratio
CN: SARS-CoV-2-infection-naïve
COVID-19: Coronavirus disease 2019
CR: SARS-CoV-2-infection-recovered
CRAE: Central retinal arteriolar equivalent
CRVE: Central retinal venular equivalent
C19-YRS: COVID-19 Yorkshire Rehabilitation Scale
DVA: Dynamic retinal vessel analysis
DVC: Deep vascular complex
DC: Deceleration capacity
ED: Endothelial dysfunction
EBV: Epstein–Barr-virus
FACS: Fluorescence activated cell sorting
FSS: Fatigue severity scale
FAZ: Foveolar avascular zone
FMD: Flow-mediated dilation
GAD-7: Generalized anxiety disorder scale-7
ICU: Intensive care unit
ME/CFS: Myalgic encephalomyelitis/chronic fatigue syndrome
OCT: Optical coherence tomography
PBMC: Peripheral blood mononuclear cells
PCR: Polymerase chain reaction
PCS: Post-COVID-19 syndrome
PHQ-9: Patient Health Questionnaire
PI: Principal investigator
PICS: Post-intensive care syndrome
PROM: Patient-reported outcome measurement
ROC: Receiver operating characteristics
RVA: Retinal vessel analysis
SARS-CoV-2: Severe acute respiratory syndrome coronavirus type 2
SOP: Standard operating procedures
SVA: Static vessel analysis
SVC: Superficial vascular complex
TMPRSS2: Transmembrane protease serine subtype 2
TLR: Toll-like receptor
WHO: World Health Organization
