# Correction: All eyes on PCS: analysis of the retinal microvasculature in patients with post-COVID syndrome—study protocol of a 1 year prospective case–control study

**DOI:** 10.1007/s00406-025-02125-6

**Published:** 2025-10-08

**Authors:** Timon Kuchler, Renate Hausinger, Matthias C. Braunisch, Roman Günthner, Rebecca Wicklein, Benjamin Knier, Nathalie Bleidißel, Matthias Maier, Andrea Ribero, Maciej Lech, Kristina Adorjan, Hans Stubbe, Konstantin Kotliar, Uwe Heemann, Christoph Schmaderer

**Affiliations:** 1 Department of Nephrology, Klinikum rechts der Isar, School of Medicine, Technical University of Munich, Ismaninger Str. 22, 81675 Munich, Germany; 2 Department of Ophthalmology, Klinikum rechts der Isar, School of Medicine, Technical University of Munich, Ismaninger Str. 22, 81675 Munich, Germany; 3 Medizinische Klinik und Poliklinik IV, LMU University Hospital Munich, Ziemssenstraße 5, 80336 Munich, Germany; 4 Medizinische Klinik und Poliklinik IV, LMU University Hospital Munich, Ziemssenstraße 5, 80336 Munich, Germany; 5 Department of Psychiatry and Psychotherapy, LMU University Hospital Munich, Nußbaumstraße 7, 80336 Munich, Germany; 6 Medizinische Klinik und Poliklinik II, LMU University Hospital Munich, Marchioninistraße 15, 81377 Munich, Germany; 7 Aachen University of Applied Sciences, Heinrich-Mussmann-Str. 1, 52428 Jülich, Germany

**Correction: European Archives of Psychiatry and Clinical Neuroscience (2023) 274:1847–1856** 10.1007/s00406-023-01724-5.

In this article the author's name Konstantin Kotilar, were incorrect but it should have been read as Konstantin Kotliar.

The original article has been corrected.

