# Supplementary material for: All eyes on PCS: analysis of the retinal microvasculature in patients with post-COVID syndrome—study protocol of a 1 year prospective case–control study
Source: Eur Arch Psychiatry Clin Neurosci. 2023 Dec 2;274(8):1847–56. doi: 10.1007/s00406-023-01724-5 (PMC11579198; doi:10.1007/s00406-023-01724-5)
Supplement: Supplementary file 1 — Supplementary file1 (DOCX 5164 KB) [file 406_2023_1724_MOESM1_ESM.docx]

**Supplementary Information: All Eyes on PCS – Analysis of the retinal microvasculature in patients with post-COVID syndrome – Study protocol of a one-year prospective case-control study**

Timon Kuchler^1^, Renate Hausinger^1^, Matthias C. Braunisch^1^, Roman Günthner^1^, Rebecca Wicklein^2^, Benjamin Knier^2^, Nathalie Bleidißel^3^, Matthias Maier^3^, Andrea Ribero^1,4^, Maciej Lech^4^, Kristina Adorjan^5^, Hans Stubbe^6^, Konstantin Kotilar^7^, Uwe Heemann^1^, Christoph Schmaderer^1^

Authors are affiliated with: ^1^ “Technical University of Munich, School of Medicine, Klinikum rechts der Isar, Department of Nephrology, Ismaninger Str. 22, 81675 Munich, Germany“; ^2^ “Technical University of Munich, School of Medicine, Klinikum rechts der Isar, Department of Neurology, Ismaninger Str. 22, 81675 Munich, Germany“; ^3^ “Technical University of Munich, School of Medicine, Klinikum rechts der Isar, Department of Ophthalmology, Ismaninger Str. 22, 81675 Munich, Germany“; ^4^ LMU University Hospital Munich, Medizinische Klinik und Poliklinik IV, Ziemssenstraße 5, 80336 Munich, Germany“;

^5^ „ LMU University Hospital Munich, Department of Psychiatry and Psychotherapy, Nußbaumstraße 7, 80336 Munich, Germany“;^6^ „ LMU University Hospital Munich, Medizinische Klinik und Poliklinik II, Marchioninistraße 15, 81377 Munich, Germany“;^7^ „ Aachen University of Applied Sciences, Heinrich-Mussmann-Str. 1, 52428 Jülich, Germany “;

Corresponding Authors:

Christoph Schmaderer, MD, Christoph.schmaderer@mri.tum.de

Timon Kuchler, MD, timon.kuchler@mri.tum.de

**Online Resource 1**

**SOP: Dynamic and static retinal vessel analysis (DVA and SVA)**

- Ask subjects/patients again for specific contraindications (glaucoma, epilepsy, cataract, pregnancy).
- Start PC: Open program for static measurement (blue button) and enter the study ID.
- Patient on chair, one eye (if possible, for comparability always the left one). 2-3 drops of mydriatic (note: after that no driving possible), wait approx. 5 minutes if no reaction drip again (2-3 drops).
- Eye patch on the eye that is not analyzed.
- First the static measurement will be described:
- Ask patient to position himself on chin rest and forehead rest (pay attention to straight position).
- Pull back the upper part of the fundoscope and present the left (or right) eye: two white clamps must be positioned in the center of the eye.
- Move the upper part of the fundoscope slightly closer to the eye, always keeping the joystick straight, until the patient can see a green light.
- When examining the left eye, press right button. The green light moves to the left. In the fundoscopy, the macula now moves to the right on the screen .
- Two white luminous points appear
- The two points must be brought on top of each other with fine joystick movements and moved into the brackets.
- The device automatically takes a picture.
- Check for quality. This is indicated by color coding (green good, red bad).
- Take at least three images of a good quality. If no qualitative measurement is technically possible (restless subject, lens opacity, ptosis, hypersensitivity, etc.), note in the protocol
- Open program for dynamic measurement, enter patient ID
- Always click **on video recording**
- Briefly explain the procedure to the patient again; flicker phases can be unpleasant, caution carefully before each flicker phase during the examination.
- Remove the cap from the front of the device; patient leaves the eye patch on
- Ask the patient to position himself on the chin rest and forehead support (make sure that he is in a straight position).
- Turn the light up slightly to about 1/3 of the intensity (if the image is too dark, turn it up carefully, but first check the position of the green light cone in the center of the subject's cornea).
- Come close with the overpart, hold joystick straight with hand until the patient sees a needle
- Advance until vessels are visible, if out of focus, turn the slider to the right or left to focus **Notion**: please do not operate the slider at all during the measurement. If the image becomes blurred, check subject position (firmly on forehead and chin) and have him/her blink.
- Ask the patient whether he sees the needle tip sharp. otherwise move the needle carefully until the sharp appears to the subject.
- Vessel marking:

Select the measurement area by the fixation needle so that the largest possible area of the superior temporal vascular street fits in on the image: N. opticus for the right eye at the bottom right, for the left eye - at the bottom left. In this way, if necessary, the measurement distances can be selected differently for a follow-up measurement, and distant vessels can also be remeasured.

The measurement should always be in the first temporal-superior are; if not possible then temporal-inferior. Vessel marking should be done 0.5 - 2 times of the optic disc diameter away from the optic nerve, as straight as possible and not over any vessel branches (Fig1).

- When program has detected both vessels, start measurement.
- During measurement: The hand is always on the joystick (to follow potential patient movements).
- Encourage patient to keep eyes open, not to move and to fixate on needle.
- End of measurement, check quality (How many flicker phases and images are recorded), save the video.

**Figure 1**: **Example image and vessel markings of the left and right fundus.** Images display ideal vessel marking. Measured sections are about 1.5 - 2 mm or about half a papilla diameter long , are not over a branch or vessel crossing and are in the ideal distance to the optic nerve.


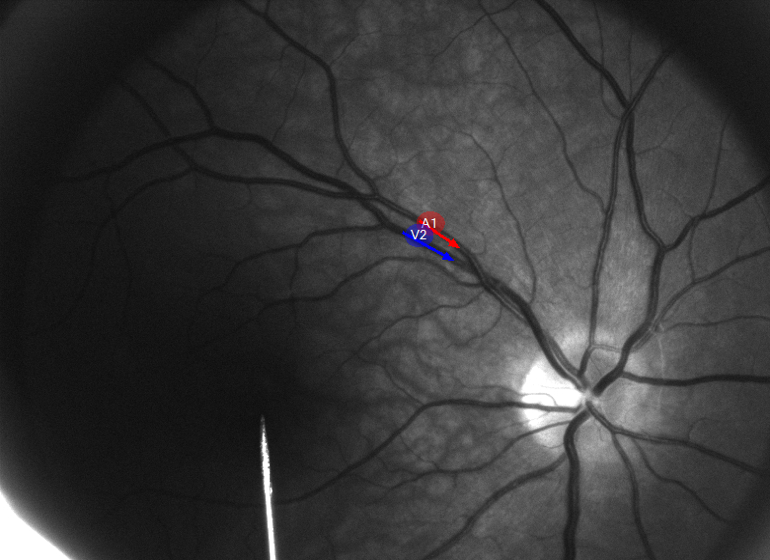

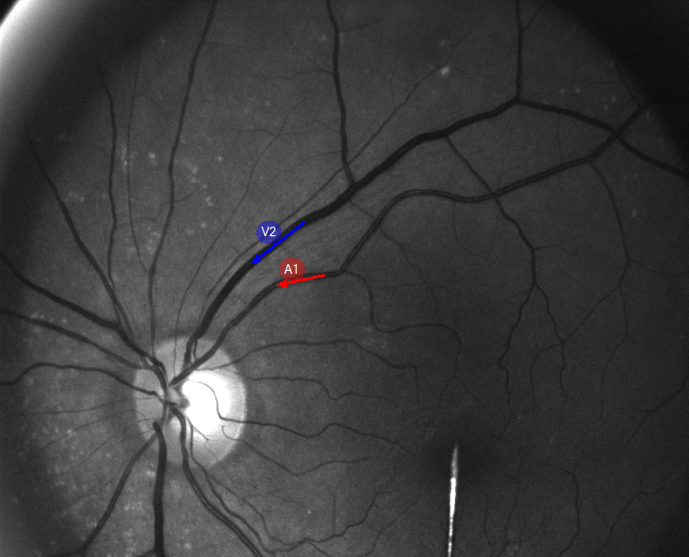


**Online Resource 2**

**SOP: Optical coherence tomography (OCT) and OCT angiography (OCTA).**

- Check if patient informed consent is signed and if patient has no further questions concerning OCT or OCTA exam.
- Start OCT/A program and enter patient ID.
- Ask patient to position himself on chin rest and forehead rest (pay attention to straight position). Adjust chin rest and table height if necessary.
- Turn off the light.
- Take of lense protection.
- Move the laser closer to visualize B-Scan in the right field in the upper 50% of the window. Adjust laser position and focus to ensure sufficient signal strength and prevent tilting or defocus artifacts.
- For OCT analysis, ask patient to watch green cross that is located on the contralateral side for peripapillary ring scan and peripapillary retinal volume measurements.
- For macular volume and OCTA analysis ask patient to watch green cross in the middle.
- Select follow-up setting if patient has been examined before.
- Check for sufficient quality (OCTA: Q value > 30) before recording.
- Record respective scans by pushing on the middle button on the joystick.
- Save images.
- Check segmentation of all images and correct manually if necessary.

**Online Resource 3**

**SOP: Adaptic optics (AO)**

- Start PC: Open AO software connected to rtx1 camera for measurement and enter the study ID.
- Patient on chair, one eye in mydriasis (if possible, for comparability always the left one).
- First the measurement will be described:
- Ask patient to position himself on chin rest and forehead rest (pay attention to straight position and adjust if necessary).
- Enter the spheric equivalent of the patient’s eye into the respective field (e.g. -2.5 diopters).
- Select “retina vessels” on the computer display.
- Pull back the rtx1 camera with the joystick and present the left (or right) eye: four white dots must be positioned in the center of the pupil.
- Move the rtx1 camera slightly towards to the eye, always keeping the joystick straight, until the four dots appear clearly on the screen.
- Ask the patient to focus on the middle of the yellow cross which appears when the rtx1 camera is positioned correctly.
- A live image of the retina will appear on the display of the computer.
- Use the scale in the middle of the computer screen to adjust the focus. The focus should be adjusted until the retinal vessels appear clearly defined.
- Select the first quarter of the upper vascular arch which can be easily marked on the computer software, so that each measurement is comparable. Take a picture using the button in the middle of the joystick. Take a picture using the button in the middle of the joystick.
- Afterwards, select the first quarter of the lower vascular arch which can be easily marked on the computer software, so that each measurement is comparable. Take a picture using the button in the middle of the joystick. Take at least three images of a good quality. If no qualitative measurement is technically possible (restless subject, lens opacity, ptosis, hypersensitivity, etc.), note in the protocol.
- Always check for quality. This is indicated during the measurements by color coding (green good, orange medium, red bad).
- Click to export the pictures into the analysis software. Fill in the axial length of the eye in the respective field (e.g. 24 mm). Select the region of interest (ROI) and analyze (Fig1). The selected region has be a straight vessel.


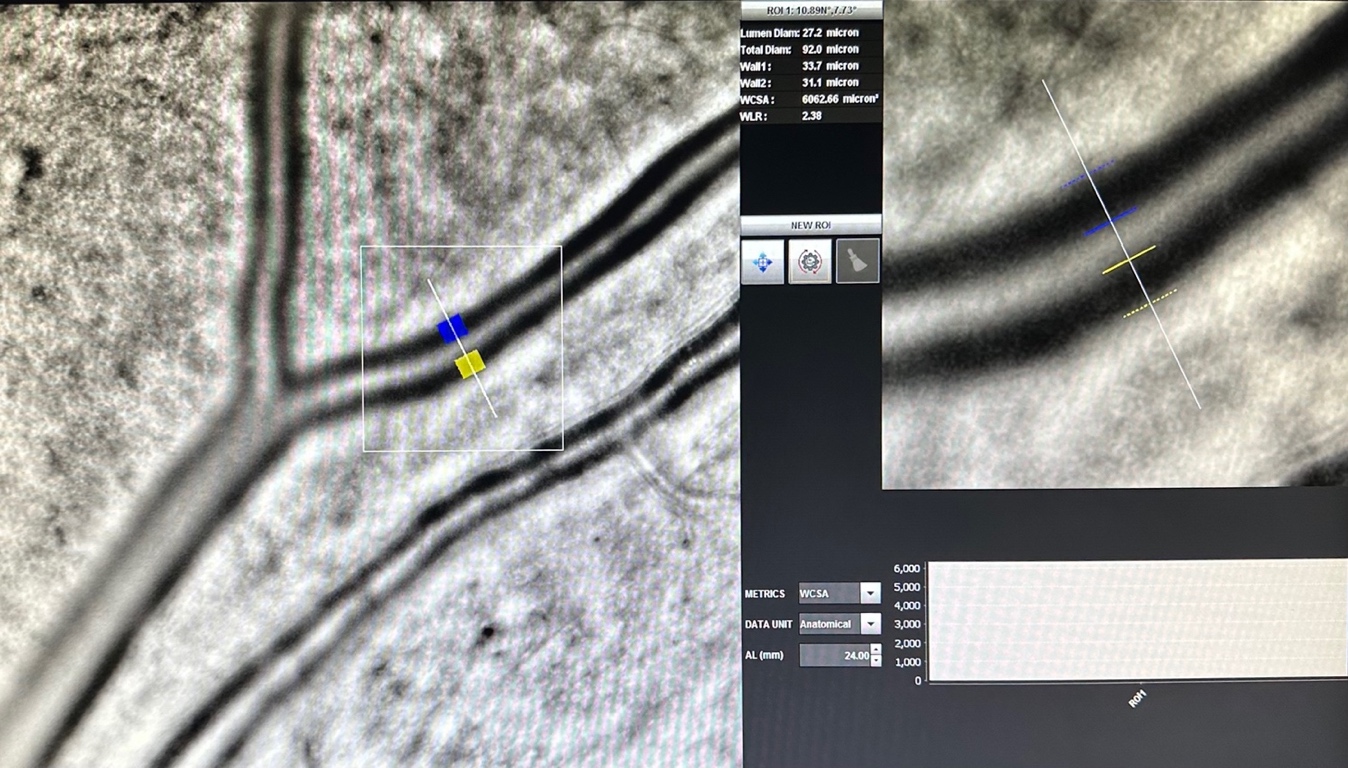


**Figure 1. Picture of retinal vessels taken with the rtx1 camera after export to the analysis software.** Selected ROI with its specific parameters (lumen diameter, total diameter, wall 1 and 2, wall-to-lumen ratio (WLR), wall cross-sectional area (WSCA)).

**Online Resource 4**

**SOP: Handgrip strength test**

Note: Before use, the handgrip dynamometer should be cleaned with alcohol tissues.

- Give clear instructions to the patient/participant e.g.:” Sit down and bend your underarm to an angle of 90 degrees. Your arm is not allowed to touch the trunk. When I say “squeeze”, you may squeeze as hard as you can until I say “stop”. There will be no give in of the trunk, the strength of your grip is directly displayed on the screen. The measurement will be repeated four times, two times now and two times after 30 minutes. If you have any pain or discomfort the measurement will be stopped immediately.”
- Give a short demonstration on how to perform the measurement.
- Ask the preferred hand and any physical limitations of the hand that might affect the strength.
- Start the test: participant needs to sit up straight the elbows at 90° angle and the wrist in a neutral position. Adjust the width of the handle to a comfortable size for the participant.
- For repeated measures always use the exact same position
- Press the ON button, the pre-programmed standard protocol should appear (5 repeated measures), write down whether the test was performed with the left or right hand.
- Start the test and write down the values after competition (Excel Sheet Handgrip strength test)
- Repeat the test after 30 minutes.
- Clean the dynamometer after completing the measurement.

**Online resource 5**

**Data entry of clinical data**

Double-Data-Verification: Each entry into the master table must be done by two independent persons. Person 1 enters the values into the table and places his abbreviation behind the file name when saving (e.g. PCS Tablle_ (for Max Mustermann). Person 2 also enters the values into the table and puts his abbreviation behind the file name when saving. Afterwards the two tables are compared by one of the supervisors (using the Excel formula: =IF (A1=B1; "identical"; "not identical").

If individual entries show "not identical", these values must be checked again. If there is any ambiguity or disagreement regarding the value entered, the supervising PI or the chief investigator must be consulted.

To track who entered the data (Person 1 or Person 2), the name must be entered in the column "Data entry Person 1" and "Data entry Person 2" in the row where the data entry took place.

All variables are pre-coded and can be found in the worksheet "Baseline T0". The coding and entry of the values must be done according to the predefined way (e.g. 1=yes, 0=no).

Likewise, an explanation of all abbreviations can be found. If there is any ambiguity regarding the coding, please consult the supervising physician or the study director immediately.

Note: All data from an examination day must be entered into the table and controlled within one week.

**Coding of questionnaires**

Several different questionnaires are used in this study to measure disease activity (e.g., PHQ9, FSS). The worksheets coding xxx (e.g. PHQ9) must not be processed. These contain the coding for the respective calculators of the questionnaires and provide incorrect results if the coding sheets are changed. Each answer to a questionnaire must be entered individually. To facilitate this, drop-down menus have been prepared, which allow for the selection of the correct score. Alternatively, a value of 0, 1, 2, 3, or 4 can be manually entered. If a value is entered that does not correspond to the predefined drop-down menu, an alert will appear to indicate an error. If a question is answered incorrectly or left unanswered, a value of "zero" should be used. The patient's ID should be entered in the first column of the calculator worksheet. The results from the respective calculator will be linked to the patient's row in the first sheet (e.g., Baseline T0), but it is important to double-check that the values appear correctly in the first sheet. At the end of the table, there is a column where comments can be entered. Please make sure that any comments are written clearly and concisely, so that they can be easily understood by other researchers.

Note: It's extremely important to enter each value carefully and accurately. If there is any uncertainty or confusion, please consult with the supervising PI or chief investigator

**Online Resource 6**

**Ethics approval of the Technical University Munich**

CC.: PD Dr. med . Christoph Schmaderer
Munich, 06.09.2022

Our reference: 2022-317-S-SR (please specify in correspondence)
Consultation according to § 15 Berufsordnung for physicians in Bavaria

Study title: Investigation of retinal vascular responsiveness in patients with post COVID-syndrome . A prospective observational study.

Applicant: Timon Kuchler

Dear Mr. Kuchler

the Ethics Committee has reviewed your application of 07.06.2022 on the basis of the documents submitted and reviewed.The Ethics Committee raises no objections to the conduct of the study. The ethical and legal responsibility for the conduct of this study remains with you.Changes in the organization and procedure are to be submitted to the ethics committee for reconsideration.

Aspects of data protection law in research projects are only examined cursorily by the Ethics Committee. This vote/assessment does not replace the consultation of the responsible company or official data protection officer.
In the case of cooperation with other institutions, the ethics committee generally recommends a contractual arrangement. For further advice, please contact the department responsible for contracts. The management of the Klinikum rechts der Isar expects data security for studies funded by public authorities or foundations, the management of the Klinikum rechts Isar expects data security to be ensured by the medical data integration center MeDIZ . Therefore, in these cases, please contact the Institute for Medical Informatics, Statistics and Epidemiology at [www.mediz.med.tum.de](https://deref-gmx.net/mail/client/xl_EYDsmUvo/dereferrer/?redirectUrl=http%3A%2F%2Fwww.mediz.med.tum.de). Please note that this vote is only valid for physicians and scientists working in the area of responsibility of the ethics commission of the TUM.

Yours sincerely

Prof. Dr. Georg Schmidt
